# Neurodegenerative Diseases: From Molecular Basis to Therapy

**DOI:** 10.3390/ijms232112854

**Published:** 2022-10-25

**Authors:** Luisa Agnello, Marcello Ciaccio

**Affiliations:** 1 Department of Biomedicine, Neurosciences and Advanced Diagnostics, Institute of Clinical Biochemistry, Clinical Molecular Medicine and Clinical Laboratory Medicine, University of Palermo, 90127 Palermo, Italy; 2 Department of Laboratory Medicine, University Hospital “P. Giaccone”, 90127 Palermo, Italy

Neurodegenerative diseases (NDs) are a heterogeneous group of complex diseases characterized by neuronal loss and progressive degeneration of different areas of the nervous system [1,2].

NDs represent a significant health problem worldwide, with an increasing incidence rate. Although the exact pathogenesis of NDs remains unclear, a complex interaction among genetic, epigenetic, and environmental factors has been proposed. To date, no effective therapeutics have been developed to slow, halt, or prevent any NDs. Thus, information on the molecular mechanisms underlying the pathogenesis of NDs is strongly sought after.

This Special Issue titled “Neurodegenerative Diseases: From Molecular Basis to Therapy” collected papers discussing advances in the field of NDs, including multiple sclerosis (MS) and Parkinson’s disease (PD), focusing on the underlying pathobiological mechanisms.

PD represents the second most common neurodegenerative disorder worldwide after Alzheimer’s Disease. In recent decades, significant advances have been achieved in the field of PD research. In an interesting review, Fonseca Cabral et al. described the emerging role of genetic variants, epigenomic modifications, and microbiome in the onset and progression of PD [3]. The exploration of these molecular aspects in an integrative and multidisciplinary manner is pivotal to the rise of personalized medicine. 

In an experimental PD model, Fieblinger et al. showed that the inhibition of caspase-3 prevented the loss of dendritic spines and long-term depression in spiny projection neurons without interfering with the ongoing degeneration of nigrostriatal dopamine neurons [4]. Thus, these findings support a possible role of caspase-3 in PD pathogenesis. 

Recently, Rab1 emerged as a promising candidate for therapeutic strategies [5]. It belongs to the family of GTPases, which are involved in regulating membrane traffic. Interestingly, Rab1 has been linked to alfa-synuclein toxicity in PD. In a cellular model of PD and human samples, it was overexpressed in surviving nigral neurons, highlighting its protective role in PD. Rab1 expression/function could be modulated by small molecules interacting with selective regions of this protein. Thus, there is an ongoing search for therapeutic approaches modifying Rab1-dependent α-syn toxicity. 

Another emerging therapeutic target is the dopamine D3 receptor (D3R). In PD experimental models, it has been shown that D3R-signalling promotes disease progression by favouring neuroinflammation and by stimulating the pathogenic CD4+ T cell response. Moreover, microglial activation is suppressed in D3R-deficient mice. Noteworthily, molecules able to block D3R attenuated the cerebral inflammation and, consequently, slowed the progression of PD. According to such evidence, Broome et al. evaluated the effect of the anxiolytic drug buspirone, a potent D3R antagonist in an experimental PD model [6]. The authors showed that buspirone had a neuroprotective effect and improved mitochondrial function and antioxidant activities. These findings encourage further investigation of buspirone in PD. 

Research has also focused on natural compounds as an alternative treatment for PD. Recently, chicoric acid (CA), a polyphenolic acid extracted from chicory and echinacea, which has antiviral, antioxidative, and anti-inflammatory activities, has been tested as a candidate in experimental studies. Wang et al. showed that CA prevented dopaminergic neuronal lesions, motor deficits, and glial activation in PD mice, along with increments in striatal brain-derived neurotrophic factor, dopamine, and 5-hydroxyindoleacetic acid. Furthermore, it regulated immunological response by reducing the levels of proinflammatory cytokines [7]. The authors hypothesised that the neuroprotective effect could be related to the manipulation of CA on the brain–spleen and brain–gut axes in PD. Further studies are warranted to confirm the beneficial effect of CA and to elucidate the underlying mechanisms. 

Hepp-Rehfeldt et al. investigated the potential beneficial effect of luteolin, a flavonoid present in many fruits and vegetables, on the central nervous system [8]. Specifically, the authors evaluated the antioxidant and anti-inflammatory activities of the glycosylated form of luteolin, known as luteolin-7-O-glucoside (Lut7), in an in vitro human neurodegenerative model (SH-SY5Y cells induced with 6-OHDA) in both undifferentiated and differentiated cells. They found that Lut7 has several neuroprotective effects, especially, a high antioxidant capacity. 

In recent decades, the neurotoxic effect of mercury has emerged in several studies, but little evidence is available on the negative effect of inorganic mercury (IHg) species, which have been detected in both contaminated food and cells of central nervous system origin. IHg represents one of the main targets of mercury associated with the neurological symptomatology of mercurial poisoning. Bittencourt et al. explored the effects of long-term exposure to IHg in adult rats’ cerebellum by evaluating the proteomic profile associated with the motor dysfunction outcome, including molecular, biochemical, and morphological approaches [9]. The global proteomic profile revealed several molecules involved in different biological processes, such as synaptic signalling, energy metabolism, and nervous system development, with all being associated with increased cytotoxicity and apoptosis and leading to poor motor coordination and balance. Thus, these findings reveal new molecular mechanisms involved in mercury toxicity. 

Another important research area includes multiple sclerosis. The aetiology of MS is still not fully understood, although the involvement of several mechanisms has been proposed. Among these proposed mechanisms, excitotoxicity has emerged as the mechanism behind the excess glutamate, the main excitatory neurotransmitter. Glutamate-induced neuronal degeneration is mainly mediated by N-methyl-D-aspartate (NMDA) receptors, and it is characterised by the formation of reactive oxygen species (ROS) and the activation of both caspase-dependent and caspase-independent cell death. Dąbrowska-Bouta et al. evaluated the effect of memantine, the uncompetitive NMDA receptor antagonist, on the modulation of neurological deficits and oxidative stress in experimental models of MS [10]. The authors showed that the pharmacological inhibition of ionotropic NMDA glutamate receptors has several beneficial effects, including an improvement in the physical activity of rats, a reduction in neurological deficits such as paralysis of the tail and hind limbs, and the modulation of oxidative stress. These findings provide evidence of a new possible therapeutic strategy for MS treatment.

In an exciting review, Van Schaik et al. described the multifaceted roles of fibronectin in MS pathogenesis and discuss promising therapeutic targets and agents to overcome fibronectin-mediated inhibition of remyelination [11]. 

Finally, in recent decades, miRNA emerged as promising tools in several clinical conditions, including MS. Pietrasik et al. described the current state of knowledge on miRNA panel expression in the different forms of MS [12].
